# Therapeutic effect of intense pulsed light on different types of chalazion in children

**DOI:** 10.1038/s41598-024-54161-2

**Published:** 2024-02-13

**Authors:** Jiao Jiang, Xiaoge Yang, Feifan Du, Wei Zheng, Yang Yang

**Affiliations:** 1 Department of Ophthalmology, Hebei Children’s Hospital, Shijiazhuang, 050000 Hebei China; 2 Department of Ultrasonic, The Second Affiliated Hospital of Hebei Medical University, Shijiazhuang, 050000 Hebei China

**Keywords:** Intense pulsed light, Chalazion, Cystic chalazion, Granulomatous chalazion, Diseases, Eye diseases, Eyelid diseases

## Abstract

This study aims to investigate the effectiveness of Intense Pulsed Light (IPL) therapy for chalazion treatment while also exploring potential variations in sensitivity among different types of chalazion. A total of 149 patients were selected to receive tobramycin combined with IPL treatment and tobramycin combined with hot compress. The treatment groups were divided into cystic type and granulomatous type according to different clinical manifestations. The course of treatment was 3 weeks. The improvement was based on the ultrasound measurement of the masses reduction of more than 50% or disappearance. In the IPL group, 17 (22.67%) cases were cured, 39 (52.00%) were effective, and 19 (25.33%) were ineffective. This includes: cystic type was cured in 3 (15.79%), effective in 5 (26.32%) cases, ineffective in 11 (57.89%) cases; granulomatous type was cured in 14 (25.00%) cases, effective in 34 (60.71%) cases, ineffective in 8 (14.29%) cases. In the hot compress group, 5 (6.76%) cases were cured, 16 (21.62%) cases were effective and 53 (71.62%) cases were ineffective. The cystic type was cured in 2 (8.00%) cases, effective in 3 (12.00%) cases and ineffective in 20 (80.00%) cases; the granulomatous type was cured in 3 (6.12%) cases, effective in 13 (26.53%) cases and ineffective in 33 (67.35%) cases. The cure rate and efficacy rate of IPL treatment is higher than that of hot compress treatment, the treatment effect of IPL treatment on granulomatous chalazion is better than that on cystic type.

## Introduction

Chalazion, characterized by easy recurrence and multiplicity, is a prevalent ophthalmic disease in children. The commonly used conservative treatment^1–6^, including antibiotic eye drops, local hot compress or physiotherapy, has a long treatment time and a low cure rate. Surgical treatment can clearly incise and curettage the formed chalazion, but pediatric surgery must be performed under general anesthesia, which may impose psychological burden on both parents and children; local injection of glucocorticoid in the lesion area also demonstrates a favorable therapeutic effect; however, injection therapy is often met with significant resistance among pediatric patients, particularly when administered via the palpebral conjunctival surface. If children are uncooperative, there is a higher risk of surgery, which may result in ocular trauma, increased intraocular tension or other risks.

Intense Pulsed Light (IPL) is an incoherent, high-intensity, pulsed light with a wavelength range of 500–1200 nm generated by xenon and capacitor banks. IPL finds extensive application in the field of skin beauty. Recent studies conducted by numerous scholars^7–10^ have demonstrated the significant efficacy of IPL in treating ocular surface diseases such as meibomian gland dysfunction, dry eye, blepharitis, and associated conjunctival diseases. The advantages of IPL treatment include short operation time, minimal trauma, and low pain levels. With its more and more extensive application in the treatment of ocular surface diseases, IPL may offer a novel approach for managing chalazion in children. This study aims to investigate the effectiveness of Intense Pulsed Light (IPL) therapy for chalazion treatment while also exploring potential variations in sensitivity among different types of chalazion.

## Object and methods

### Research object and group

A total of 149 patients diagnosed with chalazion and treated at Hebei Children's Hospital between June 2021 and December 2022 were included in the study. A single chalazion in a single eye was selected for each patient, resulting in a study group comprising 149 eyes. These eyes were randomly assigned to either the IPL treatment group (75 eyes) or the hot compress (HC) treatment group (74 eyes). In this study, chalazion was further divided into two subgroups according to the clinical manifestations of different disease courses: cystic type and granulomatous type. The child's guardian was informed about the study, agreed to participate, and provided voluntary signed consent. Inclusion criteria: (1) Fulfilling the diagnostic criteria of chalazion. (2) No prior medical treatment received since the onset of the disease. (3) Under 12 years of age. (4) Absence of ocular examination findings or other inflammatory diseases. Exclusion criteria: (1) History of meibomian gland surgery. (2) Facial or systemic skin diseases. (3) Autoimmune diseases such as systemic lupus erythematosus. (4) Photosensitive diseases or taking photosensitive drugs. (5) Patients with epilepsy. (6) Inability to cooperate with treatment due to various reasons.

### Diagnostic criteria

1. Painless eyelid masses and on obvious incentive.
2. Cystic type: circular or quasi-circular masses palpable in the eyelid, with a clear border, smooth surface and no adhesion to the skin, dark red congestion on the corresponding surface of the palpebral conjunctiva, no obvious rupture on the skin surface and eyelid conjunctiva.
3. Granulomatous type: ① Dark red cyst eminence visible on the skin's surface, the surface layer of the skin is weak or accompanied by ulcer, the formation of subcutaneous granuloma; ② Papillary eminence observed at the meibomian gland opening; ③ Local rupture of the lid conjunctiva resulted in granuloma fungoid.

### Treatment methods

1. Hot compress treatment group: tobramycin eye drops were applied to the affected eye (one drop at a time, three times daily) combined with local hot compress on the affected eyelid. The hot compress was performed using sterile gauze or towel soaked in warm water (temperature slightly higher than body surface temperature, ranging from 38 to 42 ℃), applied locally to the affected eye. This procedure was conducted twice daily for 5 to 10 min each time.
2. IPL treatment group: tobramycin eye drops combined with IPL treatment, using the “EYESIS light pulse MOPT treatment instrument for meibomian gland dysfunction (MDC)” for IPL treatment. The children were placed in the supine position, a ceramic eye mask was used to cover both eyes, and coupling agent was applied to the lower eyelid and temporal side. After wearing the protective mirror, the clinician held the treatment handle and performed the IPL treatment from the nasal side of the lower eyelid to the temporal side, administering five irradiations per eye. The pulsed light energy ranged between 8 and 12 J/cm^2^, initially starting at a lower energy level, which was adjusted based on individual tolerance. Treatment sessions were conducted once a week for a total of three repetitions.

The children were followed up after the treatment to see if there was any adverse reaction.

### Evaluation of efficacy

The duration of the treatment regimen for both groups was 3 weeks. The maximum diameter of chalazia were measured and recorded by color Doppler ultrasound before and after treatment.

1. Cure: the chalazion resolved completely, without any palpable nodules on either eyelid, and no signs of conjunctival congestion were observed on the palpebral surface.
2. Effective: the cyst or granuloma was significantly reduced after treatment, and the length of the cyst was reduced by 50% or more as confirmed by color Doppler ultrasound.
3. Ineffective: there were no significant changes in the size and shape of the mass before and after treatment, and the length of the mass changed within 50% as measured by color Doppler ultrasound, or chalazion worsening including mass enlargement, increase in number, disease progression.

### Statistical analysis

Statistical software SPSS26.0 was used for analysis. Statistical data were expressed as n (%). Chi-square test or Fisher's exact test was used to compare data between different groups. Quantitative data were expressed as mean and standard deviation, and analysis of variance (ANOVA) and least significant difference (LSD) was used to compare components. P < 0.05 indicated a statistically significant difference.

### Ethics approval

This study was performed in line with the principles of the Declaration of Helsinki. Approval was granted by the Medical Ethics Committee of Hebei Children's Hospital.

## Results

### Gender, age and disease duration of patients with different types of chalazia who received IPL and hot compress treatment

In the IPL group, there were 19 cases of cystic type (6 males and 13 females, mean age 3.68 ± 1.77 years, mean duration 2.05 ± 1.27 weeks) and 56 cases of granulomatous type (21 males and 35 females, mean age 3.34 ± 1.65 years, mean duration 6.73 ± 3.46 weeks). In the hot compress group, there were 25 cases of cystic type (13 males and 12 females, mean age 2.80 ± 1.19 years, mean duration 2.60 ± 1.76 weeks) and 49 cases of granulomatous type (20 males and 29 females, mean age 3.24 ± 1.79 years, mean duration 6.39 ± 3.41 weeks). There was no significant difference in gender and age between all groups (*P* > 0.05). The disease duration in the four groups was significantly different (*P* < 0.05). Based on LSD multiple comparisons, the cystic chalazion (including IPL and hot compress groups) exhibited a shorter disease course compared to the granulomatous chalazion group, corresponds to the clinical manifestations of chalazion. (Table 1).

**Table 1:** Comparison of age, gender and duration differences between the four groups.

	IPL-cyst	IPL-granuloma	HC-cyst	HC-granuloma	Total	χ2	*p*
Age (years)	3.68 ± 1.77	3.34 ± 1.65	2.80 ± 1.19	3.24 ± 1.79	–	–	0.346
Female	13 (68.42)	35 (62.50)	12 (48.00)	29 (59.18)	89 (59.73)	2.211	0.530
Male	6 (31.58)	21 (37.50)	13 (52.00)	20 (40.82)	60 (40.27)		
Duration (week)	2.05 ± 1.27	6.73 ± 3.46	2.60 ± 1.76	6.39 ± 3.41			0.00
Total	19	56	25	49	149		

### Comparison of the therapeutic effect between the IPL treatment group and the hot compress treatment group

**Figure 1:**
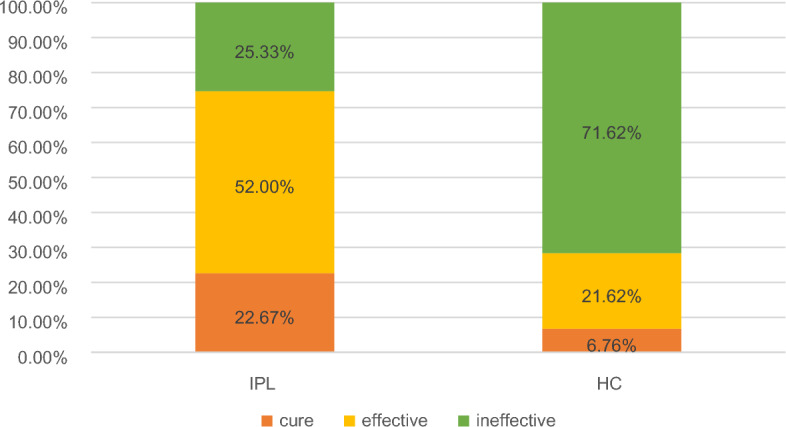
Results of IPL treatment and hot compress treatment.

There were 75 cases in the IPL group, of which 17 (22.67%) were cured (the mean diameter measured 4.31 ± 1.73 mm prior to treatment and 0 ± 0 mm post-treatment), 39 (52.00%) were effective (The mean diameter measured 4.72 ± 2.16 mm prior to treatment and 1.84 ± 0.83 mm post-treatment), and 19 (25.33%) were ineffective (the mean diameter measured 3.92 ± 1.68 mm prior to treatment and 4.22 ± 1.67 mm post-treatment). There were 74 cases in the hot compress treatment group, of which 5 (6.76%) were cured (the mean diameter measured 4.63 ± 2.05 mm prior to treatment and 0 ± 0 mm post-treatment), 16 (21.62%) were effective (the mean diameter measured 4.42 ± 2.16 mm prior to treatment and 2.16 ± 0.95 mm post-treatment), and 53 (71.62%) were ineffective (the mean diameter measured 4.18 ± 1.86 mm prior to treatment and 4.41 ± 2.17 mm post-treatment) (Fig. 1).

After treatment, the cure rate and efficacy rate of the IPL group were higher than that of the hot compress group, the difference was statistically significant (*P* < 0.05) (Table 2). No adverse reactions were found in any of the treatment groups.

**Table 2:** Comparison of therapeutic effect between IPL group and HC group.

		IPL	HC	Total	χ^2^	*P*
Therapeutic effect	Cure	17 (22.67)	5 (6.76)	22 (14.77)	32.214	0.000
	Effective	39 (52.00)	16 (21.62)	55 (36.91)		
	Ineffective	19 (25.33)	53 (71.62)	72 (48.32)		
Total		75	74	149		

### Comparison of the therapeutic effect of IPL treatment and hot compress treatment on different types of chalazia

1. Statistical results
  IPL group: 3 (15.79%) cases of cystic type cured, 5 (26.32%) effective, 11 (57.89%) ineffective; 14 (25.00%) cases of granulomatous type cured, 34 (60.71%) effective, 8 (14.29%) ineffective. Hot compress group: 2 (8.00%) cases of cystic type cured, 3 (12.00%) effective, 20 (80.00%) ineffective; 3 (6.12%) cases of granulomatous type cured, 13 (26.53%) effective,33 (67.35%) ineffective (Fig. 2).

**Figure 2:**
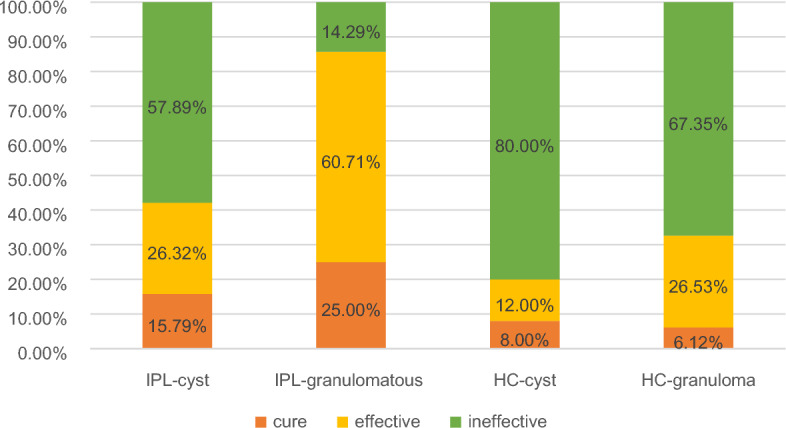
Results for the four treatment groups.
2. The treatment effect of IPL on granulomatous chalazion was better than that on cystic chalazion, and the cure rate and effective rate of the granulomatous type were higher than those of the cystic type, the difference was statistically significant (*P* < 0.05) (Table 3).

**Table 3:** Comparison of the therapeutic effects of IPL in the treatment of cystic and granulomatous chalazion.

		IPL-cyst	IPL-granulomatous	Total	χ^2^	*P*
Therapeutic effect	Cure	3 (15.79)	14 (25.00)	17 (22.67)	14.409	0.000
	Effective	5 (26.32)	34 (60.71)	39 (52.00)		
	Ineffective	11 (57.89)	8 (14.29)	19 (25.33)		
Total		19	56	75
3. There was no significant difference in the therapeutic effect of hot compress treatment on cystic and granulomatous chalazion (*P* > 0.05) (Table 4).

**Table 4:** Comparison of the therapeutic effect of hot compress on cystic and granulomatous chalazion.

		HC-cyst	HC-granulomatous	Total	χ^2^	*P*
Therapeutic effect	Cure	2 (8.00)	3 (6.12)	5 (6.76)	2.073	0.355
	Effective	3 (12.00)	13 (26.53)	16 (21.62)		
	Ineffective	20 (80.00)	33 (67.35)	53 (71.62)		
Total		25	49	74
4. There was no significant difference between IPL treatment and hot compress. treatment for cystic chalazion (*P* > 0.05) (Table 5).

**Table 5:** Comparison of the therapeutic effect of IPL therapy and hot compress therapy on the cystic chalazion.

		IPL-cyst	HC-cyst	Total	χ^2^	*P*
Therapeutic effect	Cure	3 (15.79)	2 (8.00)	5 (11.36)	2.542	0.281
	Effective	5 (26.32)	3 (12.00)	8 (18.18)		
	Ineffective	11 (57.89)	20 (80.00)	31 (70.45)		
Total		19	25	44
5. For granulomatous chalazion, the therapeutic effect of IPL was better than that of hot compress. The cure rate and efficacy rate of IPL were higher than that of hot compress, and the difference was statistically significant (*P* < 0.05) (Table 6).

**Table 6:** Comparison of the therapeutic effect of IPL treatment and hot compress treatment on granulomatous chalazion.

		IPL-granulomatous	HC-granulomatous	Total	χ^2^	*P*
Therapeutic effect	Cure	14 (25.00)	3 (6.12)	17 (16.19)	31.417	0.000
	Effective	34 (60.71)	13 (26.53)	47 (44.76)		
	Ineffective	8 (14.29)	33 (67.35)	41 (39.05)		
总计		56	49	105

## Discussion

**Figure3:**
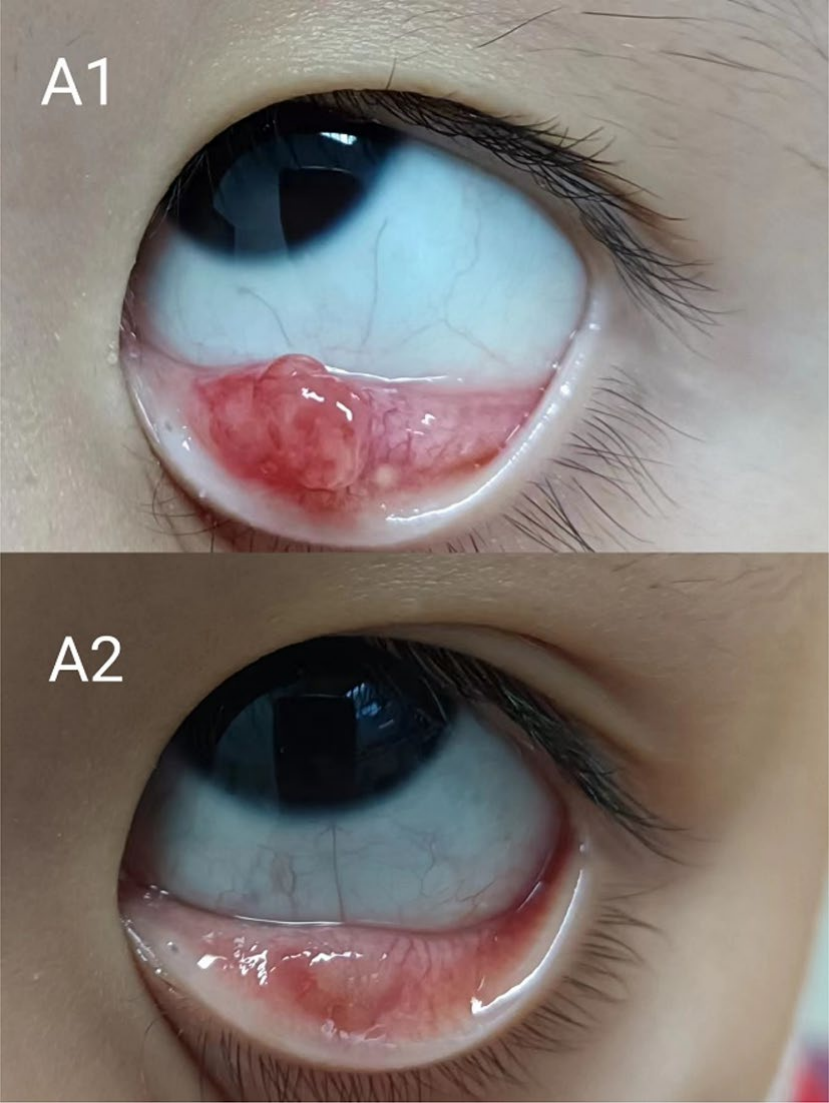
A 4-year-old female. Changes in chalazion in lower eyelids before (**A1**) and after (**A2**) IPL. (**A1**) Lower eyelids. 8 mm of chalazion. (**A2**) Chalazion much improved.

Chalazion is a chronic aseptic inflammatory granuloma that forms after obstruction of the meibomian gland ducts and accumulation of secretions in the gland^11^. Treatment options include conservative management, local glucocorticoid injection and surgical incision^1^. Many researchers have carried out in-depth studies of different treatments for chalazion^2–6^. Wu AY et al.^2^ found that tobramycin eye drops combined with hot compress reduced cysts by more than 50% in 45% of patients. In our study, utilizing tobramycin combined with hot compress resulted in an improvement rate (effective rate + cure rate) of 28.38%. The reason for this difference may be the poor compatibility of eye drop application and hot compress treatment for children in this study. Gowalla et al.^3^ showed that the cure rate of chalazion after triamcinolone injection and surgery was 84% and 87% respectively. In Nabie R's study, the success rates of intravitreal triamcinolone and surgery were 61.5% and 84% respectively. In our study, IPL treatment achieved an improvement rate reaching as high as 74.67%, significantly surpassing Wu AY's conservative approach while closely resembling the efficacy observed with invasive hormone therapy and surgery. The results showed that IPL treatment has a significant effect on the chalazion (Fig. 3).

Chalazion usually grow slowly and painlessly^11^, which is difficult to detect in its early stages. Especially for children, most patients are treated in hospital for local swelling caused by concomitant infection. The exact time of onset is difficult to trace, and the course of cysts can only be assessed by clinical physical examination. In this study, chalazion was divided into cystic type and granulomatous type according to different morphological characteristics. The treatment effect of IPL for granulomatous chalazion was superior to that for the cystic type, with an improvement rate as high as 85.71%, which was similar to the success rate of surgical treatment. There is no difference between hot compress treatment of cyst type and granulomatous type. At the same time, there was no significant difference in the therapeutic effect between IPL and hot compress for cystic chalazion, while for granulomatous chalazion, the therapeutic effect of IPL was significantly superior to hot compress. In general, IPL has a significant effect on the granulomatous chalazion. The reason for this result may be related to the mechanism of IPL therapy.

IPL energy is delivered in the form of pulses, light is absorbed by different target organs and converted into heat, and selective photothermal action avoids non-specific thermal damage in the treatment area^12^. IPL can destroy the abnormal blood capillaries of the chalazion and reduce the release of inflammatory mediators, thereby reducing the chronic inflammatory response^13,14^_._ The temperature of palpebral sebum can reach 35–38 ℃, and palpebral sebum can be successfully discharged from meibomian gland after liquefaction, However, the heating effect on the glands is temporary and it is difficult to achieve continuous blepharon drainage. Many studies have shown that Demodex infection may be one of the risk factors for chalazion in children^15,16^, and IPL can kill demodex through instantaneous high temperature^17^.

Different treatment methods have their own advantages and disadvantages. Although the effectiveness of antibiotic eye drops combined with hot compress treatment for chalazion is limited, it offers the benefits of minimal trauma, fewer adverse reactions, and convenient operation, making it a preferred initial choice for diagnosis. Local glucocorticoid injection and surgery are used in patients who do not show significant resolution of masses after a period of conservative treatment^2,11^. A study by Dhaliwal and Bhatia^18^ showed that incision and curettage of chalazion is an effective treatment option for all chalazia, particularly complex cases near the lacrimal ductule or eyelid margin. However, surgical resection, especially of recurrent and multiple chalazia, can result in palpebral conjunctival and skin scarring^19^, and resection of larger masses can easily result in the absence of meibomian glands, leading to chronic meibomian gland dysfunction (MGD)^20^. No adverse reactions were observed during IPL treatment in this study or previous studies on ocular surface disease using IPL^7–10^.

There are still some shortcomings in our study. Firstly, the sample size of this study is small, which may lead to bias caused by various factors. Therefore, the results of this study need to be further verified by large-scale, randomized controlled and multicentre prospective studies. Secondly, in our study, chalazia were observed according to different forms of clinical physical examination, which is highly subjective and leads to greater risk of misjudgment. Hence, more appropriate differential diagnosis approaches should be explored in future research endeavors. Additionally, color Doppler ultrasound was utilized with high precision to assess treatment efficacy in our study. Subsequently, by integrating information such as echo and blood flow within the lesion using ultrasound imaging techniques, it becomes feasible to determine specific subtypes of chalazion.

## Conclusions

In conclusion, the results of our study suggest that IPL treatment is effective for children with chalazia, especially the granulomatous type. For newly diagnosed children with chalazia, antibiotic eye drops combined with IPL can be chosen as treatment. After treatment, surgical treatment may be chosen depending on the prognosis.

## Data Availability

The datasets used during the current study are available from the corresponding author on reasonable request.
